# Evaluating Canine Positioning Relative to Facial Landmarks: A Study on the Accuracy of the Inner Canthus and Alar of the Nose as Reference Points

**DOI:** 10.1155/ijod/8746060

**Published:** 2025-09-15

**Authors:** Deema Ammar Quzeih, Rasha A. Alamoush, Sanaa Aljamani, Kifah Dafi Jamani

**Affiliations:** ^1^Department of Fixed and Removable Prosthodontics, School of Dentistry, University of Jordan, Amman 11942, Jordan; ^2^Department of Dentistry, The University of Jordan Hospital, Amman 11942, Jordan; ^3^Department of Restorative Dentistry, School of Dentistry, University of Jordan, Amman 11942, Jordan

**Keywords:** alar of the nose, canine position, facial landmarks, inner canthus of the eye, occlusion

## Abstract

**Background:** Accurately placing canine teeth in the dental arch is critical in achieving optimal esthetic and occlusal harmony, especially in edentulous patients. However, there is a lack of scientifically validated information to accurately place it in its true position.

**Objective:** This study aimed to verify whether the inner canthus and alar of the nose reference points align exactly with the canine cusp tip position for both genders, providing valuable insights to enhance esthetic treatment outcomes.

**Methods:** A total of 400 dental students aged between 20 and 30 years were enrolled in this study. A standardized frontal-view image of each participant was taken using a Lenovo computer camera that was set in a particular position throughout the process. The collected data was analyzed using Adobe Photoshop CSS software to determine the canine position, and the distance between the alar line and canine cusp tip line (ACT) was measured, as well as the distance from the inner canthus line to the canine cusp tip line (ICT) for both sides. Statistical analysis was performed using the independent *t*-test and chi-square test at a significance level of *p* < 0.05.

**Results:** The alar of the nose line is located distally from the tip of the canine, indicating no precise alignment with the canine cusp tip, with a significant difference on both sides (*p* < 0.001). It was seen that the inner canthus line was located mesially, approximately 2 mm away from the canine cusp tip line, with no significant differences between genders (*p* > 0.05), and the same observation was seen for the right and left sides.

**Conclusion:** The inner canthus of the eye and alar of the nose do not serve as a precise guide for positioning the canine cusp tip.

## 1. Introduction

Oral health significantly impacts the overall well-being and self-esteem of the patients [1]; it can even affect their day-to-day social interactions, especially in edentulous individuals who may experience emotional and functional distress due to changes in appearance [2]. In recent years, the demand for dental esthetics has massively increased, and has been implemented in the minds of this generation, causing more awareness of the social benefits of having a pleasing smile, which is mostly driven by social media [3]. Consequently, middle-aged and older adults are also becoming increasingly concerned with maintaining a youthful dental image [4]. This presents a striking challenge to clinicians in accurately positioning the maxillary anterior teeth to match each patient's facial features, especially in cases of complete dentures and full-mouth rehabilitations, a task historically delegated to dental technicians.

Among all teeth, the canine stands out for its fundamental role in both function and esthetics, and is frequently recognized as the cornerstone of the dental arch [5]. Furthermore, placing the canine tooth in the correct position and achieving optimal alignment can help establish proper width for the remaining anterior teeth, which in turn complements the patient's facial features [6]. Wehner et al. [7] initially suggested that an estimation of the vertical central axis of the upper canine teeth could be obtained through the extension of two parallel lines from the lateral surface of the alar of the nose directly onto the labial surface of the occlusal rims. Another approach was suggested that involves the use of a specially designed plastic measuring caliper that facilitates the measurement of the width of the nose, which will serve as a guide for determining the needed width of artificial teeth [7]. In 1986, Hoffman conducted a study to investigate the relationship between interalar and intercanine widths [8]. His findings revealed a moderate correlation (*r* = 0.413) and proposed that multiplying the interalar width by 1.31 could predict the width of the upper anterior teeth [8]. Similarly, Lee [9] in 1962, analyzed that the distance of the alar of the nose from the outer surface was equivalent to the distance between the tips of the maxillary canines. Additionally, Boucher and Wilson have proposed using the nasal width as a reference for determining anterior tooth dimensions and canine position [10, 11]. Despite these efforts, no standardized protocols currently validate their reliability, revealing a gap in the literature.

The inner canthus of the eye has also been suggested as a reference point for positioning the canine, as indicated by the Laestadius et al. [12] study in 1969. The study found that 78% of the population reaches an inner-canthus distance (ICD) plateau by their first year of age. Subsequently, the growth rate in that region shows a slower pace compared to the outer orbital dimension [12]. In a study including 240 subjects from the Indian population, it was found that the ICD could potentially serve as an initial marker for estimating the intercanine width (ICnW) of the six anterior maxillary teeth [13]. The average ICD measured was 33.19 mm in males and 31.75 mm in females. Meanwhile, the average ICnW was 53.51 mm in males and 50.73 mm in females. Therefore, the average multiplier to estimate ICnW from the mean ICD for males and females was 1.61 and 1.59, respectively [13].

Despite their long-standing use, reference points such as the inner canthus of the eye and the ala of the nose are still regarded as dogmas, which are beliefs lacking strong empirical evidence [14]. Therefore, this study aims to evaluate the validity of using these reference points to position the canine cusp tip, explore potential gender-based variation, and examine differences between the right and left sides for each participant. The research hypothesis was that the alar line and the canine cusp tip line (ACT) align, as well as the inner canthus line and the canine cusp tip line (ICT) align, on both sides of each participant, with gender differences present. The findings may help establish more evidence-based standards for prosthodontics and esthetic dental treatments, addressing a notable gap in the current literature.

## 2. Materials and Methods

The Institutional Review Board (IRB) at Jordan University reviewed and approved the present study under approval number 44-2023. A total of 400 adults Jordanian population (185 males and 215 females; aged 20–30 years) were enrolled in this study. This specific age group was selected because, according to the World Health Organization, tooth wear is a common dental condition in older adults—particularly those aged 65 and above [15]. By focusing on individuals aged 20–30, we aim to establish a reference based on a healthy dentition, free from age-related conditions such as tooth wear, and provide patients with an esthetic tooth setup that reflects the natural appearance typical of younger adults. All participants possessed six fully erupted natural maxillary anterior teeth with a horizontal overlap ranging from 1 to 3 mm and a vertical overlap that accounts for 20%–30% of the mandibular incisor height. Any participant who had a previous orthodontic treatment and rhinoplasty was eliminated, in addition to patients with dental anomalies like crowding, rotations, severe malalignment, microdontia, or even an occlusal cant. Also, any fractures in anterior teeth, artificial crowns, diastema, and proximal restorations on anterior teeth.

**Figure 1:**
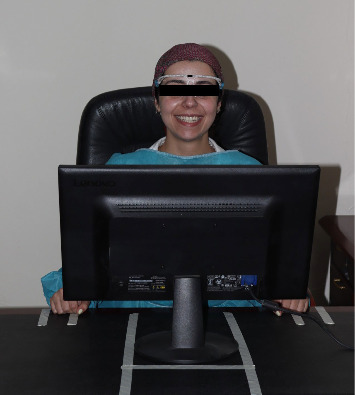
Standardization is achieved by marking designated areas for the candidate's chair and the computer's position.

**Figure 2:**
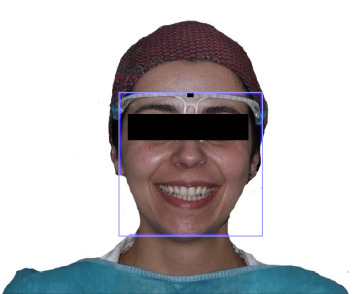
Measuring device worn, and head positioned according to the camera build-in composition guide, aligning with the upper screen border.

**Figure 3:**
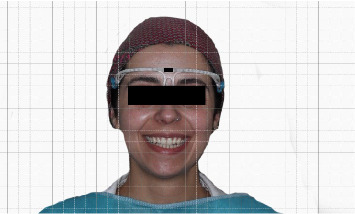
Images transferred to Photoshop for detailed analysis.

Each participant sat face forward and in a standardized position with their head oriented toward the front and their face aligned using the camera's built in composition guide (Figure 1). To ensure consistency and control image magnification, adhesive tape markers were placed on the desk and floor to guide both the chair participant position. The computer camera, fixed in a position on the monitor, remains unchanged throughout all images captured. The participant was instructed to exhibit an exaggerated smile, ensuring the exposure of the maxillary anterior teeth till the canine, and they were also directed to elevate their heads until the upper border of the composition guide aligned with the upper computer screen. In addition, to facilitate the precise conversion of distances into mm, each participant wore a measuring frame during the photography session with a marked 10 mm distance (Figures 2 and 3). The images were input into image processing software for measurement analysis (Adobe Photoshop CSS;2020), enabling the photographs of the facial parameters to be calibrated and measured with precision to the nearest hundredth (0.01 mm), providing a high level of accuracy and enhance the overall quality image. A single examiner was responsible for recording all information and performing all needed measurements to maintain consistency and minimize any potential bias in data collection and analysis.

After uploading the image, measurements were taken for the distance between the canine cusp tip line and the inner canthus of the eye (ICT) for both sides, as well as the distance between the alar of the nose line and the canine cusp tip line (ACT) using the program's measurement tools. Additionally, upon uploading the image to the software, the calibrated frame marked at 10 mm was documented within Photoshop, facilitating precise conversion of the measured distances. This process eliminates any potential distortion that could have arisen during the image-capture and upload processes. A test-retest reliability assessment was conducted on a pilot sample of 40 participants. The evaluation involved the same group of participants and setting on two different occasions to ensure consistency in administration and methodology. The correlation coefficient was calculated on the two main measurements of the study. The first measured the distance from the right ala of the nose line to the maxillary canine cusp tip line, scoring a correlation coefficient of 0.829. The second measurement, on the left side, yields a correlation coefficient of 0.953. All scores showed a high correlation, suggesting good test–retest reliability and indicating consistent methodology.

**Figure 4:**
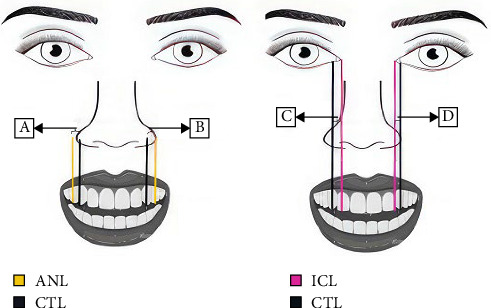
(A, B) Measured distance from CTL (canine cusp tip line) to (alar nose line) ANL. (C, D) Represent the measured distance from CTL to ICL (inner canthus line).

Statistical analysis was conducted using SPSS (version 26), and a significant level of *α* = 0.05 was set for all tests. A chi-square test was applied to assess and count the proportion of participants with aligned versus misaligned landmarks and to determine whether the proportion differed significantly between the right and left sides individually for all participants. The mean distance from ICT and from the ala of the nose line to the canine cusp tip line (ACT) was calculated separately for the right and left sides (Figure 4). An independent *t*-test (*α* = 0.05) was then used to compare the mean values between males and females for each side individually; these analyses were performed only in cases where alignment was not present, to evaluate the magnitude of deviation between groups.

## 3. Results

A chi-square test was conducted to assess the precise alignment of the inner canthus line with the canine cusp tip line. Results showed that 70 participants (17.5%) fulfilled the criteria on the right side, while 84 participants (21.0%) did so on the left side. The analysis for both sides yielded a value of (*p* < 0.001), indicating that there is a significant difference in distance between participants who met the criteria and those who did not (Table 1). On the other hand, for the precise alignment of the nasal alar line with canine cusp tip (ACT), the result revealed that 50 participants (12.5%) achieved this alignment, while the remaining deviated from it. Moreover, the findings indicated a notable significant difference between the outcome of participants whose alignment precisely matched the canine and those with deviated alignment, with a value of (*p* < 0.001). The same was seen for the left alar of the nose line, with only 12 participants (3%) achieving exact alignment with the canine cusp tip line, with highly significant differences (*p* < 0.001) between the deviated and aligned participants (Table 2).

**Figure 5:**
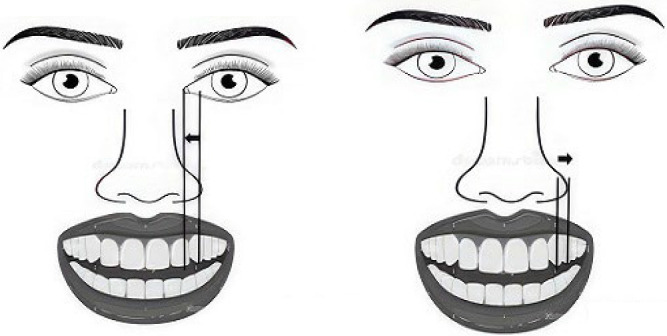
(A) Distance mesially from CTL (canine cusp tip line) to ICL (inner canthus line). (B) Distance distally from CTL to ANL (alar nose line).

In terms of the measurements, the average distance between the ICT in the distal direction (Figure 5A) was seen to be (2.3 ± 1.9 mm) on the right side, and a mean of (1.9 ± 1.9 mm) on the left side. A gender-based comparison revealed a mean distance for ICT of (2.2 ± 2.0 mm) for the right side and (2.1 ± 2.1 mm) for the left side in males, and (2.4 ± 1.94 mm) for the right side and (1.8 ± 1.8 mm) for the left side in females, with no statistically significant difference between genders (*p* < 0.09) (Table 3). Similarly, the mean distance as a whole between the alar of the nose to the canine cusp tip line (ACT) in the mesial direction (Figure 5B) is seen to be (3.1 ± 2.0 mm) for the right side and (3.9 ± 2.2 mm) for the left side. Furthermore, gender-based analysis was conducted separately for each side. For males, the mean ACT was (3.7 ± 2.1 mm) on the right side and (4.7 ± 2.3 mm) on the left side. Meanwhile, females exhibited a mean ACT of (2.5 ± 1.9 mm) on the right side and (3.3 ± 1.8 mm) on the left side. There was no significant difference observed between males and females (*p*=0.283) (Table 4).

## 4. Discussion

From the present results, the research hypothesis was rejected; neither the inner canthus of the eye nor the ala of the nose serves as a precise guide for accurately placing the maxillary canine cusp tip. In addition, no significant difference was found between genders regarding the inner canthus of the eye; therefore, an estimation of 2 mm in the distal direction can be added to position the canine tip in its true position for both genders. Additionally, no significant gender-based differences were observed between the right and left sides in the distance from the ala of the nose to the maxillary canine cusp tip (ACT). Therefore, a mesial adjustment of approximately 3–4 mm can be considered when positioning the canine cusp tip using the ala of the nose as a reference. These findings are very crucial for patients seeking full mouth rehabilitation or complete dentures without having any preoperative records. Similar to the findings of Scandrett et al. [16]., where the alar width was found to serve only as an initial guide, the present study supports the conclusion that the alar line does not reliably correspond to the actual position of the maxillary anterior teeth. Scandrett et al. [16] recommended using the alar landmark in conjunction with other facial references (intercommissural width, bizygomatic width, sagittal cranial diameter and interbuccal frenum distance) to achieve accurate anterior tooth positioning. In our study, we further observed that a mesial adjustment of approximately 3–4 mm from the alar line brings the canine cusp tip closer to its actual anatomical position, offering a more clinically applicable refinement to its use.

Additionally, the findings of this study are consistent with Srimaneekarn et al. [17] conclusions, revealing a significant difference in location between the alar line and canine cusp tip line on both sides (*p* < 0.001), Moreover, a consistent positive value was seen between these two lines, indicating that the alar line consistently appears to be positioned distally to the canine cusp tip line in both genders, mirroring the observations made in this study. However, a notable gender-based difference was seen, contrary to the findings of this study. This difference could potentially be attributed to racial variation, as Srimaneekarn et al. [17] solely focused on a specific ethnic group, the Thai population. Another study was conducted on the Thai population, which also showed no correlation between IAW (interalar width) with Intercanine tip width [6].

No previous study has quantified the distance from the inner canthus to the canine cusp tip, as was performed with this study. However, Srimaneekarn et al. [17] studied the relationship between a line extending from the inner canthi of the eye to the ala of the nose in proximity to the canine cusp tip's location. The outcome showed that the IA line (the inner canthus of the eye to alar line) cannot accurately estimate the position of the canine cusp tip on either side in both genders, as it was located more distally to the canine tip CT. Concluding a failure in the reliability of using these landmarks in canine positioning, which aligns with the findings of the current study.

Sinavarat et al. [6] reported a moderate positive correlation (*r* = 0.547) between the intercanine tip width and the distance measured from the inner canthus to the alar of the nose (DPICa). While this suggests a general linear relationship between facial dimensions and dental arch width, it does not imply that the IA line accurately locates the position of individual anatomical features such as the canine cusp tip. This distinction further supports the conclusion of the present study, emphasizing the limitation of using facial lines like the IA line as definitive guides for tooth positioning [6].

It is also important to note that the present study was conducted on a Jordanian population, whereas the referenced studies by Srimaneekarn et al. [17] and Sinavarat et al. [6] were based on Thai populations. These ethnic and anatomical variations may account for some of the observed differences in findings and must be considered when interpreting and applying these findings in different clinical contexts.

The study has some limitations, including the imposition of an age restriction between 20 and 30 years. This was primarily selected due to practical reasons; it was more feasible and efficient to obtain consent forms from dental students, in addition to them being easily accessible and readily available. Additionally, this age range was chosen to eliminate any age-related alterations in dental alignment and health. Further, a disproportionate gender participation with a higher number of females than males. This accurately reflects the genuine distribution observed among dental students. Although strict exclusion criteria were applied—including anterior restorations, prosthetic crowns, attrition, and diastemas—to control for confounding factors, this may still limit the generalizability of the findings to a broader population. Additionally, a paired *t*-test was not used to compare the right and left sides within the same patient; however, this did not impact the primary objective of the study, which was to determine whether the inner canthus and the alar of the nose can serve as reliable landmarks for canine positioning.

## 5. Conclusions

Neither the right nor the left alar of the nose can be used as a precise, reliable indicator for aligning the maxillary canine cusp tips. For practical guidance, it can be advised to add approximately 3–4 mm mesially on the right and left side when positioning the canine cusp tip using the alar of the nose as a guide.

This study revealed that the inner canthus of the eye does not precisely align with the maxillary cusp tip position. For practical guidance, it can be advised to add a 2 mm distance distally to the inner canthus of the eye line when used as a guide for canine cusp tip positioning for both genders and sides.

## Figures and Tables

**Table 1:** Frequency of having the right and left inner canthus line in alignment with the canine cusp tip line (ICT).

ICT coincidence		Frequency	Percentage (%)	Chi-square
Right ICT	Coincide	70	17.5	*p* < 0.001
	Not coincide	330	82.5	

Left ICT	Coincide	84	21.0	*p* < 0.001
	Not coincide	316	79.0	

*Note:* ICT, inner canthus with canine cusp tip line.

**Table 2:** The frequency of having the right and left alar nose line in exact alignment with the canine cusp tip (ACT).

ACT coincidence		Frequency	Percentage (%)	Chi-square
Right ACT	Coincide	50	12.5	*p* < 0.001
	Not coincide	350	87.5	

Left ACT	Coincide	12	3	*p* < 0.001
	Not coincide	388	97	

**Table 3:** Mean distance between inner canthus line (ICL) to canine cusp tip line (CTL) in mm scale.

Sex	Right	Left	*p* ^#^-Value
	Mean ± SD	Mean ± SD	
Male	2.2 ± 2.0	2.1 ± 2.1	0.09
Female	2.4 ± 1.94	1.8 ± 1.8	
*p* ^*∗*^-Value	0.481	0.096	

*Note* : *p*^*∗*^, *p*-value of independent samples *t*-test for comparison between male and female for each side alone; *p*^#^, *p*-Value of independent samples *t*-test for comparison regarding the gender. *p*-value significant at ≤0.05.

Abbreviations: CTL, canine cusp tip line; ICL, inner canthus line.

**Table 4:** Mean distance between ala nose line (ANL) to canine cusp tip line (CTL) in mm regarding gender.

Sex	Right	Left	*p* ^#^-Value
	Mean ± SD	Mean ± SD	
Male	3.7 ± 2.1	4.7 ± 2.3	0.283
Female	2.5 ± 1.9	3.3 ± 1.8	
*p* ^*∗*^-Value	<0.001	<0.001	

*Note* :  *p*^*∗*^, *p* value of independent samples *t*-test for comparison between male and female for each side alone; *p*^#^, *p*-Value of independent samples *t*-test for comparison regarding the gender. *p*-value significant at ≤0.05.

Abbreviations: ANL, ala of the nose line; CTL, canine cusp tip line.

## Data Availability

The data are provided within the manuscript.
